# The Treatment of Hysterical Aphonia

**Published:** 1894-07

**Authors:** 


THE TREATMENT OF HYSTERICAL APHONIA.—Seifert (*Arztliche Rundschau ;* November 4, 1893). The method which Seifert advises is a combination of laryngeal massage, of laryngeal compression (as advocated by Oliver), and of methodical vocal gymnastics. He begins with external manipulation by stroking over the thyroid region, extending thence over the general area occupied by the jugular vein and cervical lymph vessels, and the flow from these vessels is accelerated also by deep inspiration, causing a negative thoracic pressure. This latter inspiratory movement Seifert regards as of great importance, for he believes that in the majority of cases of hysterical aphonia there is an inco-ordination not only of the muscles of the cords, but also of the general respiratory muscles. The patients must be taught, therefore, anew the art of deep inspiration, with each stroking of the larynx.

When this result has been attained, gentle, but rapid lateral compression of the larynx is introduced. Thereby the action of the adductors is supported in that the upper and back part of the thyroid cartilage is compressed between the thumb and forefinger while the patient makes an attempt at phonation. Gerhardt endeavors to have the patient phonate not only during expiration, but also during inspiration, and claims good results from the procedure.

Finally, the patient is directed to count from one to twenty, slowly and distinctly, while the larnyx is at the same time repeatedly compressed in the manner indicated. If any number in the series is indistinctly pronounced it is repeated over and over again until perfect distinctness is reached. A deep inspiration must precede each tone.—*American Medico-Surgical Bulletin.*

